# Impact of hypoxia on the molecular content of glioblastoma-derived exosomes

**DOI:** 10.20517/evcna.2023.52

**Published:** 2024-01-11

**Authors:** Simona Di Giulio, Elisabetta Carata, Marco Muci, Stefania Mariano, Elisa Panzarini

**Affiliations:** Department of Biological Sciences and Technologies (Di.S.Te.B.A.), University of Salento, Lecce 73100, Italy.

**Keywords:** Hypoxia, glioblastoma, extracellular vesicles, exosomes, HIF-1α, noninvasive biomarker

## Abstract

Hypoxia is a pathologic condition characterized by a tissue oxygen deficiency due to either decreased oxygen intake from outside and/or disruption of oxygen utilization in cells. This condition may arise when the oxygen demand exceeds its supply or the partial pressure of oxygen is below 10 mmHg. This situation poses a significant problem for glioblastoma (GBM) patients as it can activate angiogenesis, increase invasiveness and metastatic risk, prolong tumor survival, and suppress anti-tumor immunity, making hypoxic cells resistant to radiotherapy and chemotherapy. Low oxygen levels in tumors can cause severe cellular changes that can affect the release of extracellular vesicles (EVs), especially exosomes (EXOs), altering their proteomic profile both qualitatively and quantitatively. EXOs represent an adaptive response to hypoxic stress; therefore, they can be used to determine oxygen levels in cancer and assess its aggressiveness. They not only release signaling molecules to attract cells that promote the formation of small vessel walls but also send signals to other tumor cells that trigger their migration, which in turn plays a crucial role in the formation of metastases under hypoxia. This review investigates how the molecular profile of GBM-derived exosomes changes under hypoxic conditions, offering future possibilities for noninvasive diagnosis and monitoring of brain tumor patients.

## INTRODUCTION

Low oxygen content in numerous solid tumors, including GBM, is a condition that leads to tumor development, treatment resistance, and mortality. The tumor microenvironment (TME) can be divided into three regions based on the distance of cells from the vasculature and physiological abnormalities: normoxic, hypoxic, and necrotic regions. Cells in the hypoxic region adapt to low oxygen levels because they can reorganize the microenvironment through genetic, molecular, and metabolic changes, leading to growth, metastasis, and resistance to therapy. Cellular responses to hypoxia are mainly mediated by hypoxia-inducible factor (HIF), a heterodimeric transcription factor that accumulates within cells in response to reduced oxygen levels. HIF consists of an oxygen-sensitive HIF-α subunit (HIF-1α, HIF-2α, or HIF-3α) and a HIF-1β subunit^[1,2]^, which is constitutively expressed. During hypoxia, HIF-1α protein increases in the cytoplasm and migrates to the nucleus, where it combines the HIF-1β subunit and another expression regulator to form the conserved activated hypoxia-dependent element (HRE), which is responsible for the expression of genes regulating cell viability, proliferation, epithelial-to-mesenchymal transition (EMT), angiogenesis, metastasis, and therapy resistance^[3]^. Moreover, HIFs have been shown to promote EXOs biogenesis and secretion, as well as alteration of their cargo^[4]^.

This unique type of cell communication within tumors has promising potential for clinical application in GBM treatment. Indeed, EVs are considered potential targets for GBM therapy, as numerous publications have reported that EVs play a central role in the GBM microenvironment, growth, angiogenesis, infiltration, and even diagnosis^[5]^. GBM is a prevalent and malignant form of brain tumor in adults. It is characterized by its rapid growth, lack of clear boundaries, and infiltration into surrounding tissues. Patients have a survival rate of approximately 15 months, with only a small percentage (3%-5%) surviving beyond 36 months due to the tumor’s aggressive growth^[6]^. The heterogeneity of GBM results in a complex and aggressive disease course with limited therapeutic success^[7]^.

In addition to the phenotypic, morphologic, and cellular heterogeneity features of the tumor, GBM exhibits unlimited self-renewal, higher intrinsic chemo- and radioresistance, and tumorigenic cancer stem cells, termed GBM stem cells (GSCs), which contribute to tumor initiation and rapid activation of invasive growth and metastasis even in the presence of relatively small numbers of cells^[8]^.

To promote tumor proliferation, immunosuppression, and angiogenesis, GBM cells constantly communicate with their surrounding heterogeneous microenvironment, which consists of cancer and non-cancer cells (i.e., endothelial cells, immune cells, GSCs, astrocytes) and the extracellular matrix (ECM). As in all solid tumors, the TME of GBM is severely compromised by low oxygen levels (hypoxia), which promote resistance to chemotherapy and radiotherapy, immunosuppression, cancer stem cells, and angiogenesis^[9]^.

Radiotherapy is a major approach in managing GBM progression. The clinical outcome of this approach is based on the increased radiosensitivity of actively dividing cancer cells compared to normal tissue^[10]^. This strategy aims to eradicate tumors while minimizing damage to surrounding cells through three distinct pathways: direct cell death triggered by DNA double-strand breaks, indirect apoptosis resulting from vascular damage, and stimulation of anti-tumor immune response^[11]^. High-dose irradiation can have a dual effect on tumor immunity. On one hand, it can stimulate the immune system by promoting the release of tumor antigens and activating immune cells. On the other hand, it may also lead to immunosuppression by inducing vascular damage and, consequently, blood perfusion, which in turn increases tumor hypoxia and the expression of HIF-1α, the pivotal transcription factor for maintaining oxygen balance and immunosuppression^[12]^. HIF-1α not only drives tumor cells to adapt to hypoxic conditions and proliferate but also serves as a central regulator of tumor-immune escape. This occurs through the transcriptional upregulation of a variety of genes that suppress both innate and adaptive immune responses directed against cancer cells. For instance, the recognition of neoplastic cells by the immune system relies significantly on the expression of MHC class I. While irradiation has been shown to increase MHC class I expression, hypoxia and HIF-1α have the opposite effect and suppress their levels, impairing the ability of antigen-presenting cells (APCs) to recognize tumor cells^[12]^. Therefore, inhibition of HIF-1α emerges as a potential strategy to attenuate hypoxic cell survival and proliferation, while potentiating the anti-tumor immune response^[13]^.

A study by Dai *et al.* identified an upregulation in the antisense transcript levels of hypoxia-inducible factor-1α (AHIF) in cancerous GBM cells^[14]^. Knockdown of AHIF in these cells and derived EXOs increases the apoptotic cells, thereby inducing both cell viability and invasive capability, suggesting AHIF as a potential therapeutic target for GBM^[14]^.

Notably, there is growing evidence that damage caused by ionizing radiation has a significant impact on exosome-mediated intercellular signaling, both by increasing the quantity of released vesicles and by altering their molecular content^[15]^. A recent study by Arscott *et al*. suggested that radiation-induced exosomes contained a great abundance of mRNA and proteins associated with cell motility and were found to be involved in cancer invasion^[16]^. *In vitro* irradiated GBM cell lines were also shown to induce M2-tumor promoting phenotype of microglia through exosome release. Furthermore, the M2-polarized microglia actively stimulated the growth of irradiated GBM cells *via* the CCL2/CCR2 axis^[16]^.

GBMs differ from other solid tumors in that they are shielded by the brain-blood barrier (BBB), which prevents the entry of peripheral immune cells. Nevertheless, the integrity of the BBB is compromised by inflammation, rapid tumor growth, and invasion of the tumor by immunosuppressive immune cells from the bloodstream^[17]^. GBM is classified as an “immunologically silent” subtype, characterized by low numbers of lymphocytes and high levels of a type of macrophage population, referred to as tumor-associated macrophages/microglia (TAMs)^[18]^. In addition, this immunologically silent microenvironment is provided by immunosuppressive signaling molecules, e.g., transforming growth factor beta (TGF-β), interleukin 10 (IL-10), prostaglandin E2, and other immune cells, e.g., immunosuppressive natural killer T cells (NKT), regulatory T/B cells (T/Breg), and myeloid-derived suppressor cells (MDSCs). In particular, TAMs are attracted to the TME, which induces polarization to an anti-inflammatory or pro-tumor phenotype, ultimately leading to the maintenance of tumor progression^[19,20]^.

These features drive malignancy and contribute to tumor recurrence. To achieve this goal, GBM cells utilize various means of communication with TME cell types, including direct cell interactions via membrane receptors and their ligands, as well as the transport of molecules packaged and delivered by EVs^[21]^.

EVs are lipid bilayer-enclosed nanostructures (50-1000 nm) that are secreted by both normal and tumor cells^[22]^. In 2018, the International Society for Extracellular Vesicles (ISEV) revised its extensive Minimal Information for Studies of Extracellular Vesicles (MISEV) guidelines to enhance standardization and quality in the field of EVs. As accurate categorization of EVs remains exceptionally difficult, it can routinely be based on (a) physical properties of EVs, such as size [“small EVs” (sEVs) and “medium/large EVs” (m/lEVs), with defined ranges, e.g., < 200 nm or > 200 nm] or density; (b) biochemical composition; (c) cell of origin; (d) biogenesis pathway^[23]^.

According to their biogenesis, EVs can be mainly divided into three subtypes: exosomes (EXOs, 30-200 nm), microvesicles (MVs, ectosomes or microparticles up to 1000 nm), originating from the endosomal compartment or by direct shedding of the plasma membrane, respectively, and apoptotic bodies (1-5 μm in diameter). EXOs originate from the formation of early endosomes, in which intraluminal vesicles accumulate, originating multivesicular bodies (MVBs), which eventually fuse with the plasma membrane and release ILVs into the extracellular space^[22,24]^. Both EXOs and MVs are present in a variety of human biofluids, including urine, semen, serum, lymph, saliva, tears, nasal secretions, bile, amniotic fluid, and breast milk^[25,26]^. Various bioactive compounds can be found in EVs, such as mRNAs, microRNAs (miRNAs), DNA, proteins, and lipids. These molecules can be transported to recipient cells by various mechanisms, including clathrin- or caveolin-mediated endocytosis, phagocytosis, micropinocytosis, and simple fusion with the plasma membrane. Upon delivery, the cargo can trigger an intracellular signaling cascade either through interaction with plasma membrane receptors, direct entry into the cytoplasm or during transit to the nucleus^[27,28]^.

EVs are released at all stages of disease, making them an ideal source of biomarkers for screening, early diagnosis, and therapy monitoring, as well as for improving clinical decision-making. This is of particular interest in cancer, where EVs are also emerging as a next-generation platform for liquid biopsy, in addition to disseminated cancer cells and circulating cell-free DNA. The considerable intrinsic heterogeneity observed for GBM poses a number of significant challenges, including molecular profiling, prognostic assessment, tumor progression monitoring, and evaluation of response to treatment^[29]^. These challenges are exacerbated by the invasive nature of brain surgery and tissue biopsy. It should also be noted that the informative value of a biopsy taken from a single site is limited, as it may not cover the entire tumor^[11]^. Tumors such as GBM are in a constant evolutionary process and respond to clonal selection, hypoxia, and different treatment modalities. Against this background, liquid biopsy could provide a minimally invasive, safe, and highly sensitive alternative to conventional tissue biopsies for GBM patients^[30]^. Liquid biopsy may facilitate tumor monitoring by detecting various types of biomarkers in peripheral blood or cerebrospinal fluid, through the analysis of tumor-derived circulating material^[31]^. Despite advancements in liquid biopsy continuously improving its utility in the diagnosis, prognosis, and monitoring of GBM, its application remains significantly restricted^[30]^. This is primarily attributed to the lack of standardized techniques for sample collection and processing compared to traditional tissue biopsies, consequently leading to great variability in results and their interpretation^[32]^. Moreover, while early detection of GBM is crucial for better treatment outcomes, liquid biopsy methods may not consistently detect the disease at early stages, limiting its utility in early diagnosis. However, it remains a complementary tool alongside other diagnostic and treatment modalities^[30]^.

Since cancer cells release a larger amount of EVs compared to healthy cells, circulating EVs could serve as a valuable source for insights into tumor status and disease progression^[5]^ and the utility of liquid biopsy is very important in avoiding tissue biopsies, and in the treatment of GBM, it could be an approach to overcome the limitations of invasive techniques and provide a tool for real-time monitoring of treatment and patient response, as well as information on minimal residual disease^[33]^.

Both the molecular signature and the amount of EVs released are significantly affected by hypoxic conditions. This impacts the recognition and uptake of EVs by recipient cells, which in turn may alter the biological functions triggered by EVs; in fact, the molecules driven together with the mutant proteins or other oncogenic mechanisms in cancer cells work together to enable cancer proliferation and facilitate tumor progression^[34]^.

Here, we will focus on providing a comprehensive overview of the interplay between EVs, in particular EXOs and hypoxia in GBM. We will summarize the current literature on (a) the effect of hypoxia on EXOs released by GBM in terms of quantity and content; (b) the biological role of EXOs released under hypoxia in GBM.

## ROLE OF HYPOXIA IN THE GBM MICROENVIRONMENT

Hypoxia is a pathological state in which oxygen deficiency occurs in the body when external oxygen supply is reduced and/or oxygen utilization in cells is impaired^[35]^. Cancer cells respond to this low O_2_ partial pressure by stimulating neovascularization of pre-existing blood vessels, which is an important process for tumor proliferation and metastasis and serves to transport nutrients and oxygen and remove metabolic waste from tumor cells^[36]^.

**Figure 1:**
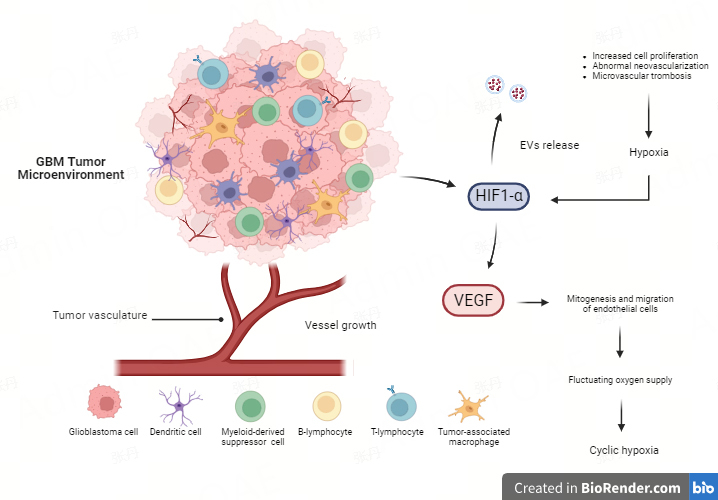
GBM tumor microenvironment. The abnormal neovascularization, due to uncontrolled tumor growth, leads to poor oxygen diffusion and hypoxic regions in the tumor mass, promoting the expression of HIF-1α. This mediator of hypoxia is responsible for the regulation of various processes, including angiogenesis, by enhancing the levels of VEGF that, in turn, act as an inducer of mitogenesis and migration of endothelial cells. This process leads to “cyclic hypoxia” characterized by a dynamic phase of hypoxia and reoxygenation within the tumor mass. Moreover, HIFs have been shown to promote EXOs biogenesis and secretion, promoting cancer metastasis and therapy resistance by mediating both the migration of glioma cells and the proliferation and migration of endothelial cells. Figure was created with https://www.biorender.com/. GBM: glioblastoma; HIF: hypoxia-inducible factor; VEGF: vascular endothelial growth factor; EXOs: exosomes.

Tumor hypoxia is a hallmark of GBM and is related to the abnormal neovascularization observed in this tumor type^[37]^. There are numerous hypoxic regions and extensive invasion in the expanding periphery of the main GBM tumor mass^[38]^. The characteristic cell structure of GBM includes pseudopalisades, resulting from the migration of tumor cells away from a central hypoxic region, leading to the formation of an invasive front^[39,40]^. Tumor growth in GBM results in inadequate oxygen supply and hypoxic regions due to neovascularization, increased cell proliferation, and microvascular thrombosis causing vascular occlusion^[37]^, as well as high hydrostatic pressure outside the GBM vasculature, which induces intratumoral edema, a significant contributor to morbidity in GBM patients^[41]^. These effects depend on the pathological features of GBM vessels, which are irregular, unstructured, extensively permeable, and easily collapsible, and have increased diameters and robust basement membranes. One of the major factors driving angiogenesis is the overexpression of vascular endothelial growth factor (VEGF), particularly VEGF-A, released in the TME under the stimulation of HIF, which triggers the upregulation of various pro-angiogenic factors^[1]^. High levels of VEGF-A stimulate the formation of leaky blood vessels to supply nutrients and oxygen to the rapidly growing tumor cells, by binding to VEGFR-1 and VEGFR-2 receptors on the endothelial cell membrane. This interaction triggers a cascade of events that stimulates mitogenesis and migration of endothelial cells^[42,43]^. In this context, altered organization and instability of the vasculature, following excessive recruitment of VEGF, lead to fluctuating oxygen supply within the tumor mass, a condition known as “cyclic hypoxia”, characterized by a dynamic phase of hypoxia and reoxygenation^[44]^ [Figure 1].

Hypoxia is a serious problem for GBM patients as it promotes cell survival and tumor metastasis progression in primary and distant brain tissues through the degradation and restructuring of the ECM, which allows tumor cell proliferation and spread from the original tumor site by creating spaces and scaffolds^[45]^. This poses a major problem not only for surgery but also for radiotherapy and chemotherapy. Oxygen status in GBM can be monitored using magnetic resonance imaging if a substantial limitation of oxygen diffusion is identified, corresponding to the absence or impairment of blood flow^[46-48]^.

## THE INTERPLAY BETWEEN HYPOXIA AND EVS RELEASE

EXOs secreted by tumor cells can be used to determine the extent of hypoxia in a tumor and to predict its aggressiveness. Specifically, EXOs derived from hypoxic GBM cells not only release signaling molecules to attract cells that regulate the formation of small vessel walls, but they also send signals to other tumor cells, triggering their migration, which plays a pivotal role in the formation of metastases^[49,50]^.

The uncontrolled division of tumor cells leads to the formation of cell clusters that are far from blood vessels and, therefore, do not receive sufficient oxygen. Normally, hypoxia is one of the factors that trigger apoptosis. However, in the case of a tumor, these conditions may lead to the selection of more viable cells, such as cells with mutations in apoptosis-associated genes. Such cells no longer respond to pro-apoptotic signals, so other deleterious mutations may accumulate in them, leading to a more aggressive behavior of such a cancerous tumor (more intense cell division, ability to form metastases, *etc*.)^[51]^. For example, a lack of oxygen in tumor cells has been shown to alter the genes responsible for cell adhesion. As a result, tumor cells can detach from neighboring cells, contributing to the formation of metastases^[52]^. Nonetheless, HIF, as the primary mediator of hypoxia, seems to play a crucial role in microvascular formation by controlling the expression of angiogenic molecules, including VEGF, which stimulates the expansion of endothelial cells and thus the creation of new vasculature in the tumor to supply it with oxygen^[53]^.

Moreover, tumor cells can positively reprogram the cells surrounding the tumor by secreting membrane vesicles, containing signals that stimulate cell division and prevent the mechanism of apoptosis.

Several studies have shown that EXOs can mediate hypoxia-dependent intercellular signaling in GBM^[54]^. These vesicles, released by hypoxic GBM cells, can facilitate cell-to-cell communication, and cause substantial alterations in gene expression in neighboring normal oxygenated tumor cells, many of which are involved in cancer infiltration and therapy resistance^[55]^. Hypoxia also induces glioma cells to secrete EVs with specific pro-angiogenic molecules, such as cytokines, growth factors, proteases, and miRNAs, affecting endothelial cells and fostering angiogenesis. In turn, these endothelial cells, altered by glioma EVs, release potent growth factors and cytokines, inducing the proliferation of pericytes, vascular smooth muscle cells, and glioma cell migration^[56]^.

**Figure 2:**
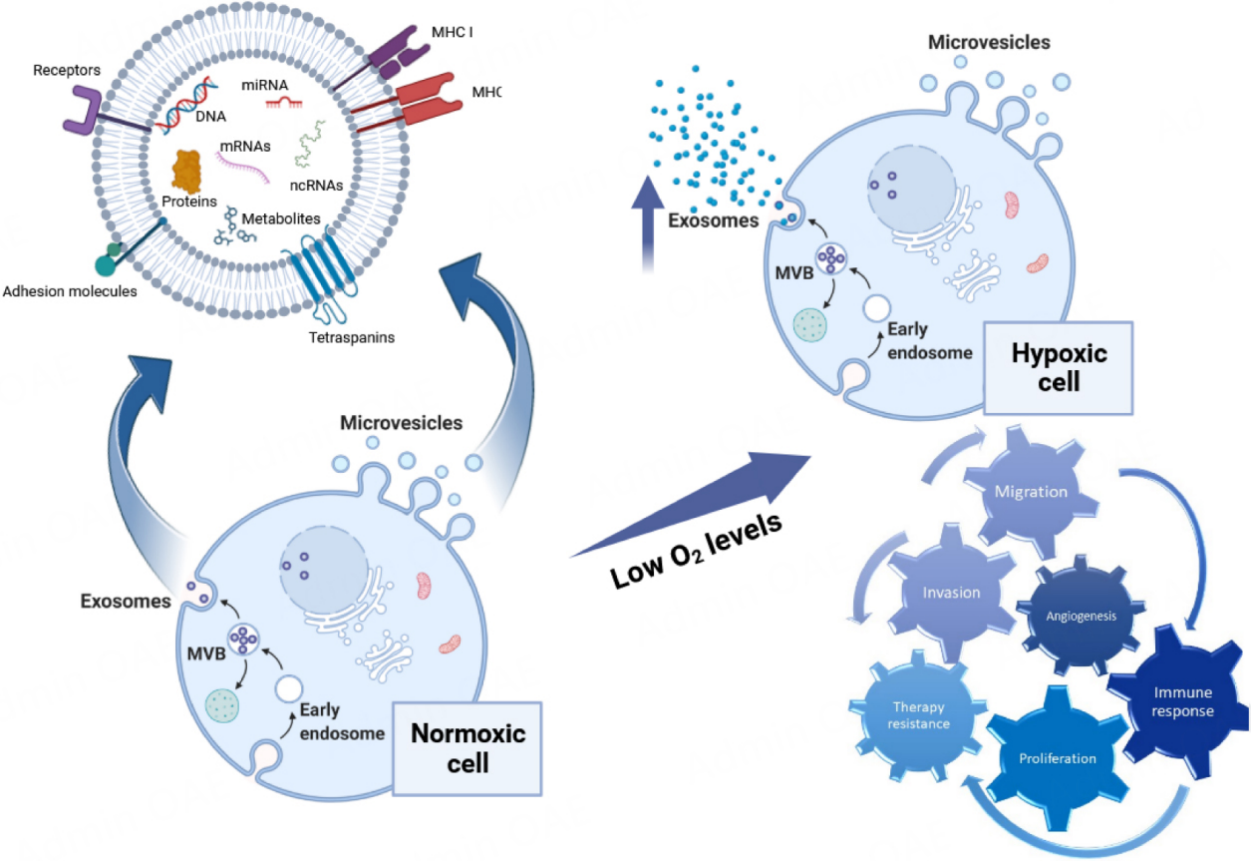
The generation of EVs and the characteristics of hypoxia-induced EVs in the recipient cells. EXOs originate within MVBs by budding into the endosomal membrane, resulting in the release of small vesicles within this compartment. MVBs fuse with the cell membrane to release EXOs into the extracellular space, while MVs form directly from the outward shedding of the plasma membrane. Both EVs types carry diverse cargos, including DNA, mRNA, ncRNA, proteins, and metabolites. In hypoxic cells, there is an increased release of exosomes, which play a multitude of roles upon reaching the recipient cells. Hypoxic EVs play roles in invasion, migration, angiogenesis, proliferation, immune responses, and drug resistance of cancer cells. Figure was created with https://www.biorender.com/. mRNA: messenger RNA; ncRNA: noncoding RNA; EVs: extracellular vesicles; EXOs: exosomes; MVBs: multivesicular bodies.

Figure 2 reports a schematic representation of the main effects of EXOs released under hypoxic conditions.

*In vitro* studies on glioma cells have unveiled a notable enrichment of hypoxia-regulated mRNAs and proteins within EXOs^[56]^. These proteins include matrix metalloproteinases, lysyl oxidase, platelet-derived growth factor, IL-8, and caveolin^[57]^. Interestingly, several of these exosome-enriched factors have been linked to unfavorable prognoses in GBM patients. A recent study demonstrated that EXOs originating from GBM cells, cultured under hypoxic conditions, possess the ability to induce angiogenesis both in *ex vivo* and *in vitro* conditions, through the phenotypic alteration of endothelial cells^[58]^. Intriguingly, hypoxic EXOs derived from GBM cells induce endothelial cells to secrete various robust growth factors and cytokines, stimulating PI3K/AKT signaling in pericytes activation and, in turn, promoting migration^[56]^. Furthermore, it was observed that EXOs derived from hypoxic conditions exhibited enhanced autocrine activation, improving the migratory behavior of GBM cells. These findings correlated with a significant increase in tumor vascularization, vascular pericyte proliferation, and GBM cell invasiveness in a mouse xenograft model, compared to exosomes from normoxic conditions^[59]^.

Increased secretion of EVs by breast cancer cell lines mediated by HIF-1α in hypoxic environments has been observed^[60,61]^. Similarly, lung cancer cells also show an increase in EXO release and higher levels of exosomal proteins such as CD9, CD81, and HSP70 under hypoxia^[62]^. In general, the TME, which includes oxygen levels, significantly influences the response of cancer cells to chemotherapy and radiotherapy^[63]^. Previous studies have demonstrated that alterations in the TME result in a modification to the proteome and transcriptome of a cell. In line with recent findings, these changes are mirrored in the content of exosomes released by cells. Glioma cells cultured under hypoxic conditions show a marked increase in HIF-1α and galectin-1 levels, both of which are typically upregulated in hypoxic environments and have cytoprotective effects^[55]^.

## HYPOXIA-DERIVED EXOSOMAL miRNAs

miRNAs are small single-stranded noncoding RNAs that act as guiding molecules in RNA silencing^[64]^. Studies have shown that miRNAs are capable of regulating different pathways, including loss of cellular identity and proliferation, and changes in the regulatory mechanisms controlling cell death, suggesting their potential involvement in cancer^[64]^.

The biogenesis of miRNAs starts in the nucleus, where DNA harboring miRNAs is transcribed by RNA polymerase II to produce primary miRNAs (pri-miRNAs). These molecules undergo processing by Drosha (double-stranded RNA-specific ribonuclease), resulting in the formation of hairpin RNAs comprising 70-100 nt. Subsequently, the hairpin pre-miRNAs are conveyed to the cytoplasm by exportin 5, where they are further processed by a Dicer (double-stranded ribonuclease). Upon maturation, double-stranded miRNAs convert into single-stranded miRNAs, and mature miRNAs are selectively packaged into EXOs^[65]^.

Recent research indicates that miRNAs contribute to the development of GBM^[66]^. Specifically, miR-21 was the first miRNA to be significantly increased in six GBM cell lines and is now recognized as a key oncogene that acts on various components of p53 and TGF-β signaling pathways in GBM cells^[67]^. Additional studies on other miRNAs that are abnormally expressed in GBM cells, such as miR-10bmiR-34a, miR-146b, miR-221, and miR-222 showed an impact on cell cycle, migration, and invasion of glioma cells, as well as on stem cell properties^[68]^. Moreover, miRNAs have also been reported to function as crucial mediators of hypoxia response, influencing the regulation of cell cycle, apoptosis, metastasis, and resistance to anticancer therapy. Some of them were found to contain HIF-1α response elements (HREs) in their promoters and were demonstrated to be under the regulation of HIF-1α^[69]^. Interestingly, a known hypoxia-regulated miRNA (HRM), miR-210-3p, exhibited strong induction in hypoxic glioma cell lines (U-87MG and U-251MG) as well as in hypoxic GBM tumor samples, indicating its utility as a marker for hypoxia or as a therapeutic target in GBM. These findings demonstrate that miR-210-3p contributes to GBM cell survival in the TME and enhances aggressiveness by conferring temozolomide resistance and targeting HIF-3α, a known negative regulator of hypoxia-inducible gene expression^[70]^. It has been demonstrated that miRNAs are packaged into GBM cells-derived EXOs [Table 1].

**Table 1:** Main miRNAs found in GBM-derived EVs

**miRNAs in GBM-derived EVs**	**Tumorigenic functions**	**Refs**
miR-21 ↑, miR-148a ↑, miR-130b-3p ↑, miR-182-5p ↑	Angiogenesis and invasiveness	[71-74]
miR-221 ↑, miR-222 ↑, miR-34a ↓, miR-146b ↓, miR-10b ↑, miR-543 ↓, miR-486-5p ↓, miR-485-3p ↓, miR-185 ↓	Tumor cell proliferation and migration	[75-81]
miR-210-3p ↑, miR-1238 ↑	Temozolomide resistance	[82,83]
miR-301a ↑	Radiation resistance	[84]
miR-10a ↑, miR-29a ↑, miR-1246 ↑	Immunosuppression	[85,86]

↑: increased; ↓: decreased.

Yue *et al*. demonstrated that exosomal miR-301a (exo-miR-301a) is selectively released by hypoxic GBM cells and is associated with HIF-1α status^[87]^. Therefore, it has the potential to function as a diagnostic and prognostic biomarker for human GBM. Moreover, the authors have shown that exo-miR-301a, produced by hypoxic GBM cells, can affect sensitivity to radiotherapy by modulating the Wnt/ β-catenin signaling pathway. To assess the expression of miR-301a, which is significantly increased in glioma tissues, and exosomal miR-301a, the authors examined the expression of HIF-1α as a biomarker of hypoxia. ELISA results showed that GBM cells exhibited a response to hypoxia with an increase in nuclear HIF-1α. The levels of all exosomal markers, such as CD9, CD63, CD81, and HSP70, were higher in hypoxic cells than in normoxic cells. Interestingly, hypoxia markedly upregulated the expression of miR-301a and exo-miR-301a, while downregulation of HIF1α resulted in a decrease in their expression^[87]^.

To explore the impact of tumor cells on endothelial cells, human umbilical vein endothelial cells (HUVECs) were plated in the presence of EXOs derived from normal human astrocytes (HA) and U-251MG and U-87MG GBM cells, in a hypoxic environment. Results showed that EXOs from hypoxic U-251MG and U-87MG cells promoted invasion and tube formation of HUVECs, endothelial permeability, and migration of tumor cells across endothelial barriers. These data suggest that hypoxic EXOs from glioma cells promote angiogenesis and disrupt the endothelial cell barrier, leading to intravasation and extravasation of tumor cells. Microarray analysis showed that the levels of miR-182-5p, miR-543, miR-486-5p, miR-485-3p, and miR-185 increased in primary cells under hypoxic conditions, and only miR-182-5p was significantly increased in glioma cell lines in comparison to HA cells. Moreover, when HIF-1α was knocked down in U-251MG and U-87MG cells, the increase in miR-182-5p levels in EXOs failed to occur under hypoxic conditions^[74]^. Considering the great importance of miRNAs in hypoxic TME, it is crucial to identify and analyze hypoxia-regulated miRNA in GBM. This may provide insights into the molecular mechanism of hypoxia resistance in GBM and potentially impact the diagnosis and treatment of the disease.

A Swedish research group investigated in detail the molecular composition of EXOs secreted by GBM cells^[56]^. One of the goals of the study was to investigate whether the composition of EXOs is related to the state of the cells, including the degree of hypoxia. For this purpose, it was necessary to determine if EXOs secreted by tumor cells under oxygen deficiency differed from those secreted by cells in normal conditions. EXOs purified from the blood plasma of patients with GBM or produced by different GBM cell lines cultured under different oxygen conditions were used. EXOs recovered from cells deprived of oxygen contained proteins important for GBM development and associated with a state of hypoxia. Furthermore, these vesicles also contained a variety of mRNAs: among the approximately 15,000 types of RNA molecules found in GBM cells, 6,500 were present in EXOs. In addition, eight different RNA molecules found in EXOs varied between hypoxic and nonhypoxic cells. These RNAs were associated with genes involved in tumor progression, i.e., lysyl oxidase (LOX), IGF binding protein (IGFPB) 3, adrenomedullin (ADM), inhibitor of DNA binding 2 (ID2), Bcl-2/adenovirus E18 19-kDa-interacting protein 3 (BNIP3), N-Myc downstream-regulated gene 1 (NDRG1), procollagen-lysine 2-oxoglutarate 5-dioxygenase 2 (PLOD2), and plasminogen activator inhibitor 1 (PAI1). Interestingly, the amount of RNA present in GBM cells was linked to the level of hypoxia, and higher levels of hypoxia were associated with a more aggressive form of the tumor^[56]^.

A recent clinical study by Graziano *et al.* shows that the chaperone Hsp60 was increased in GBM cells and the depletion *in vitro* was associated with tumor regression. Hsp60 levels are subject to regulation by molecular factors, including miRNAs^[88]^. The authors also revealed that Hsp60-related miRNAs released into the extracellular space and circulation were significantly increased in EVs isolated from the plasma of patients with malignant glioma, in a manner reflecting the status of the disease^[88]^.

## HYPOXIA-DERIVED EXOSOMAL PROTEINS

Different studies have shown that hypoxic cells produce greater amounts of EXOs compared with normoxic cells^[60]^. Additionally, over 50% of the proteome secreted by hypoxic carcinoma cells may be linked to EXOs^[89]^. These findings provide quantitative evidence for the complex role of EXOs in the hypoxic response. Interestingly, recent evidence has shown that the tumorigenic and pro-metastatic function of exosomes can be effectively antagonized by downregulation of the small GTPase Rab27a^[90]^, which has been shown to participate in exosome biogenesis.

Similar to microRNA, protein loading in EXOs has been reported to be mediated by endosomal sorting complexes required for transport (ESCRT), which are involved in the formation of multivesicular bodies, and ceramide pathways, known to be an important apoptosis-inducing factor in various tumor cells. Exosome protein composition reflects the state of the producing cells, including those associated with hypoxia. Thus, it was found that the molecular composition of EXOs reflects the degree of hypoxia state in the tumor and allows for the prediction of its aggressiveness [Table 2].

**Table 2:** Diagnostic and prognostic protein markers carried within GBM exosomes

**Protein cargos in GBM-derived EXOs**	**Tumorigenic functions**	**Refs**
MMPs, TGF-β1, PAI1, VEGF	Angiogenesis, cell proliferation and migration	[56]
PTX3, IL-8	Inflammation, angiogenesis, invasiveness	[56]
PDGFR	Tumor cell proliferation (poor prognosis)	[91]
EGFR	Resistance to apoptotic stimuli and to chemotherapy	[92]
CD26, CD44, CD133	Chemoresistance markers	[93]
CAV-1	Glioma progression, drug resistance, and invasiveness by regulating cell adhesion	[94,95]
LOX, TSP1, ADAMTS1	Tumor progression, metastasis, and angiogenesis	[55]
HSPs	Cancer progression metastasis, radio- and chemoresistance	[88,96,97]
Semaphorin 3A	Increased vascular permeability	[98]

MMPs: matrix metalloproteinases; TGF-β1: transforming growth factor-beta 1; PAI1: plasminogen activator inhibitor 1; VEGF: Vascular endothelial growth factor; PTX3: pentraxin 3; IL-8: interleuchin-8; PDGFR: platelet-derived growth factor receptor; EGFR: epithelial growth factor receptor; CAV-1: caveolin 1; LOX: lysine-6-oxidase; TSP1: thrombospondin-1; ADAMTS1: A disintegrin-like and metalloproteinase with thrombospondin type 1 motif 1; HSPs: heat-shock proteins.

In a study conducted by Kucharzewska *et al.*, GBM-derived EXOs were compared with age and sex-matched controls^[56]^. The results showed that EXOs were enriched in different hypoxia-regulated proteins that may be involved in tumor migration and invasiveness. Among these, the most notable include matrix metalloproteinase 9 (MMP9), pentraxin 3 (PTX3), IL-8, platelet-derived growth factor receptor (PDGFR), CD26 (also known as dipeptidyl peptidase-4), and plasminogen activator inhibitor 1 (PAI1)^[56]^. Increased expression of membrane raft domain-associated caveolin-1 protein (CAV1) in GBM cells and tumors compared to normal astrocytes and human brain tissue has been reported in the literature^[64,94,99-101]^. Additionally, hypoxic regions of GBM stained with glucose transporter 1 (GLUT1) also show increased expression of CAV1^[55]^. In addition, Western blotting analysis of GBM hypoxia exosome proteins revealed increased levels of lysine-6-oxidase (LOX), disintegrin, thrombospondin-1 (TSP1), A disintegrin-like and metalloproteinase with thrombospondin type 1 motif 1, (ADAMTS1), and VEGF. These proteins are known to be associated with hypoxia-induced neovascularization and metastasis, as well as vascular function/activation status^[55]^, suggesting that this exosomal protein signature may comprehensively reflect hypoxic signaling of high-grade gliomas.

The invasiveness of GBM under hypoxic conditions has also been linked to their increased production of hyaluronic acid (HA)^[101]^, which is one of the major components of the ECM in the brain. Moreover, GSCs have been identified by their marked expression of the stem marker and HA receptor, CD44, which contributes to the migratory and invasive abilities of these cells^[102]^.

The hypoxic environment stimulates GBM cells to release EVs containing proteins involved in actin cytoskeleton remodeling, ECM-receptor interaction, focal adhesion, and transendothelial migration of leukocytes, suggesting that EVs derived from hypoxic gliomas contribute to the migration phenotype in GBM cells^[103]^.

There is cumulative evidence supporting that EVs secreted by GBM cells are enriched in heat-shock proteins (HSPs), associated with cancer progression and metastasis^[96]^. Particularly, Hsp60 and Hsp70 are highly expressed in glioma cells and released enclosed into EVs under stress conditions, including hypoxia, hyperthermia, ischemia, heavy metal or ionizing radiation exposure, and infections^[104]^. Consequently, there is a notable upregulation of HSP expression in tumor cells compared to their normal counterparts. Hsp70 is a pivotal factor in controlling malignancy, cellular growth, resistance to treatments, and unfavorable prognosis. In addition, Hsp60 levels are elevated in numerous tumor types, and its increased expression plays an essential role in carcinogenesis. Hsp60 acts not only within cells but also in the extracellular environment and contributes to intercellular communication. Increased HSP expression has also been associated with tumor resistance to chemotherapy and radiation, suggesting the potential use of these proteins as prognostic or diagnostic indicators^[97]^.

## CONCLUSIONS

It is widely known that high-grade gliomas, such as GBM, display characteristics of hypoxia, intense angiogenesis, neovascularization, and resistance to chemotherapy and radiotherapy. Research has shown that GBM cells in hypoxic regions release EXOs that contain increased levels of proteins and RNA, which are associated with tumor aggressiveness. Additionally, the molecular composition of these vesicles is significantly representative of the oxygenation status and aggressiveness of GBM. In the future, these findings may lead to the use of EXOs as a minimally invasive "liquid biopsy" technique, which is rapid, cost-effective, and can identify tumors at an early stage.

## DECLARATIONS

### Copyright

© The Author(s) 2024.
